# Navigator‐Based Slice Tracking for Multiband Accelerated Liver DWI

**DOI:** 10.1002/mrm.70354

**Published:** 2026-03-24

**Authors:** Ke Zhang, Simon M. F. Triphan, Congcong Fu, Na Zhang, Christian H. Ziener, Mark E. Ladd, Heinz‐Peter Schlemmer, Hans‐Ulrich Kauczor, Oliver Sedlaczek

**Affiliations:** ^1^ Department of Diagnostic & Interventional Radiology Heidelberg University Hospital Heidelberg Germany; ^2^ Translational Lung Research Center (TLRC) German Center for Lung Research (DZL) Heidelberg Germany; ^3^ Department of Diagnostic & Interventional Radiology With Nuclear Medicine Thoraxklinik at Heidelberg University Hospital Heidelberg Germany; ^4^ Division of Radiology German Cancer Research Center Heidelberg Germany; ^5^ Paul C. Lauterbur Research Center for Biomedical Imaging, Shenzhen Institute of Advanced Technology Chinese Academy of Sciences Shenzhen China; ^6^ Division of Medical Physics in Radiology German Cancer Research Center Heidelberg Germany; ^7^ Faculty of Physics and Astronomy Heidelberg University Heidelberg Germany; ^8^ Faculty of Medicine Heidelberg University Heidelberg Germany

**Keywords:** liver diffusion weighted imaging, navigator‐based slice tracking, prospective motion compensation

## Abstract

**Purpose:**

Clinical liver diffusion‐weighted imaging (DWI) suffers from long measurement time and respiratory motion. In this study, we employ a navigator‐based slice tracking technique (NAV) to prospectively compensate for respiratory motion in multiband (MB) accelerated liver DWI.

**Methods:**

A single gradient‐echo slice readout at the location of the diaphragm along the inferior–superior direction was acquired as a navigator. Navigator acquisition and fat suppression were inserted before each transverse imaging slice of the readouts of a 2D spin‐echo EPI based DWI sequence, with 20 repeats including 32 training navigators (4 preparing scans) at the beginning of the measurement obtained in about 3 min. For comparison, MB accelerated DWI without NAV during free breathing was also acquired. To evaluate the effect of NAV on image quality, coefficients of variation (CoV), signal to noise ratio (SNR), and sharpness were calculated. Six healthy volunteers and three patients with lesions were examined at 1.5 T.

**Results:**

The motion caused by respiratory activity was effectively computed using the navigator signal. For measurement with NAV, averaged CoV was significantly lower when compared to without NAV (0.256 ± 0.086 vs. 0.262 ± 0.091, *p* < 0.001). SNR and liver sharpness were also increased. The shapes of both kidney and spleen were continuous and smooth in the coronal view with NAV. Detailed structure in different organs could be found with NAV compared to without NAV in the cyst and tumor patients.

**Conclusion:**

This study demonstrates the feasibility of a NAV technique to reduce respiratory motion in MB accelerated liver DWI.

## 1 Introduction

Liver diffusion‐weighted imaging (DWI) has become a central component in abdominal MR imaging protocols, offering a wide range of clinical information. The usually high contrast is routinely used for the detection of pathologies, but the ability to further characterize focal liver lesions [1, 2], yielding hints for the differentiation between benign and malignant makes it clinically irreplaceable. It plays an important role in monitoring treatment response in patients undergoing oncologic follow‐up particularly under systemic therapy or after local ablative treatments [3, 4, 5]. Here, the apparent diffusion coefficient (ADC) calculated from one pair of *b*‐values is of central importance. Beyond focal lesion assessment, DWI has gained attention in the evaluation of diffuse liver diseases such as non‐alcoholic fatty liver disease, hepatic fibrosis, and cirrhosis, where diffusion metrics may serve as surrogate biomarkers for tissue composition, fibrosis and disease progression [6, 7, 8].

Despite its utility, liver DWI remains technically challenging. The relatively long acquisition time and sensitivity to respiratory motion often compromise image quality, reproducibility and quantitative reliability. This is particularly true for the in‐line calculated ADC. Traditional breath‐hold approaches [9] are limited by patients capability for long breath‐hold durations, particularly in elderly patients or those with compromised respiratory function, while respiratory gating or triggering methods [10, 11] prolong scan time and introduce variability. As a result, there is a growing interest in developing DWI techniques that enable robust imaging under free‐breathing conditions without sacrificing image quality or acquisition efficiency.

Simultaneous multislice (SMS) or multiband (MB) DWI has emerged as a promising strategy to address the challenge of long scan times. By exciting and acquiring multiple slices simultaneously using multiband radiofrequency pulses, MB‐DWI significantly reduces acquisition time while maintaining signal‐to‐noise ratio and spatial resolution [12, 13, 14, 15]. However, when applied in the abdomen, especially the liver, residual motion from free‐breathing can still degrade image quality and introduce artifacts across the simultaneously acquired slices.

To overcome this limitation, motion‐compensated acquisition strategies are being explored. In this study, we integrate a navigator‐based slice tracking method [16] with MB‐accelerated single‐shot EPI DWI to enable robust free‐breathing liver imaging. Respiratory motion is continuously monitored using a projection navigator signal, which is used in real‐time to adjust the position of each subsequent slice dynamically. This motion‐adaptive approach, referred to here as NAV, aims to maintain slice alignment with the liver anatomy despite ongoing respiratory displacement. The core novelty of this work is the translation and technical integration of navigator‐based slice tracking into multiband‐accelerated free‐breathing liver DWI.

The objective of this work is to evaluate the feasibility and performance of free‐breathing liver DWI using single‐shot MB‐EPI with navigator‐based slice tracking. We hypothesize that this technique can produce high‐quality diffusion images with reduced motion artifacts and reproducible quantitative metrics, offering a clinically practical alternative to conventional gated or breath‐hold DWI protocols.

## 2 Methods

### 2.1 Subjects

This prospective study included six healthy volunteers (4 female, 2 male; mean age: 30 ± 6 years), who were examined using a 1.5 T clinical MRI system (MAGNETOM Aera, Siemens Healthineers AG, Erlangen, Germany) equipped with an 18‐channel anterior body coil combined with a posterior spine array for signal reception. In addition to the healthy cohort, three patients with lesions were included for evaluation: two patients with abdominal cysts and one patient with a known hepatic tumor. All participants provided written informed consent prior to the examination. The study protocol was approved by the institutional ethics committee and conducted in accordance with the Declaration of Helsinki.

### 2.2 Imaging Protocol and Acquisition

All MRI examinations were performed under free‐breathing conditions. The DWI sequence was based on a 2D single‐shot spin‐echo EPI, enhanced with MB acceleration. A sagittal oriented single‐slice gradient‐echo navigator acquisition was embedded before each imaging slice to monitor respiratory motion. The navigator was positioned at the level of the diaphragm and projected along the inferior–superior (IS) axis to capture respiratory displacement in real‐time.

**FIGURE 1:**
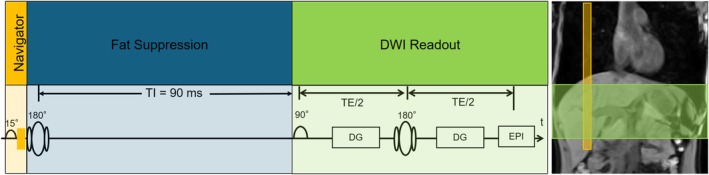
Diagram of the liver DWI sequence. Respiratory motion was tracked using a single‐slice gradient‐echo projection navigator positioned along the inferior–superior axis at the level of the diaphragm. The navigator was acquired immediately before each DWI slice, followed by fat suppression. The navigator plane (highlighted in yellow) was oriented perpendicular to the diaphragm, while the imaging volume (shown in green) was planned to cover the liver and kidneys. DG is the diffusion gradient and EPI is the echo‐planar imaging.

Navigator acquisition and spectral fat suppression using SPAIR (SPectral Attenuated Inversion Recovery) were inserted prior to each axial imaging slice, as illustrated in Figure 1. A total of 20 dynamic repetitions were acquired, including 32 training navigator projections and 4 preparatory scans at the beginning of the acquisition. The total acquisition time was 3 min and 10 s. Sequence parameters were as follows: FOV = 400 × 240 mm^3^, in‐plane iPAT factor = 2, multiband factor = 2, matrix size = 120 × 72 × 16, resolution = 3.3 × 3.3 × 5 mm^3^, slice gap = 2.5 mm, interleaved slice ordering, axial orientation, TE = 59 ms, TR = 1500 ms, TI_SPAIR_ = 90 ms, FOV_nav_ = 200 × 200 × 10 mm^3^, res_nav_ = 64, flip angle_nav_ = 15°, TR_nav_ = 4.23 ms. To reduce g‐factor, blipped‐controlled aliasing in parallel imaging (blipped‐CAIPI) was used [17].

Diffusion encoding was applied in three orthogonal directions using two *b*‐values (50 and 500 s/mm^2^). For each volunteer, two separate DWI acquisitions were performed for comparison: one using MB acceleration with navigator‐based slice tracking (NAV), and the other using MB acceleration without NAV during free breathing (FB). FB refers to free breathing, with no use of NAV, respiratory triggering, or gating. Single‐band (SB) and multiband tests were acquired separately and compared in one representative subject. Finally, a respiratory‐triggered measurement (TRIG) with a total acquisition time of about 6 min was also acquired (using SB) for comparison.

### 2.3 Navigator‐Based Slice Tracking

Motion tracking was performed using a 1D projection of the navigator signal along the IS direction. Real‐time motion estimation of the diaphragm was extracted from the navigator and used to update the position of the subsequent imaging slice dynamically [16, 18, 19, 20]. This slice‐tracking approach aimed to compensate for respiratory motion prospectively, minimizing inter‐slice misregistration and motion artifacts in free‐breathing acquisitions.

The first 32 acquired navigator echoes were used as training data. Signal voids in the navigator signal caused by saturation of the imaging slices were removed by truncating the navigator signal. This truncation position was determined from the peak of the averaged magnitude signal of the training navigators. To improve SNR, signals from receiver coils located near the diaphragm were combined. Optimal coil elements were selected using a coil clustering approach [21] applied to the training navigator data and were kept fixed for subsequent motion tracking. Respiratory motion was estimated using the Fourier shift theorem. Diaphragm displacement was derived from the phase difference of the Fourier‐transformed navigator signal. The estimated motion was transmitted in real time to the pulse sequence, providing dynamic adjustment of the imaging slice position during free‐breathing. This motion analysis and feedback was implemented to run on the scanner directly.

### 2.4 Postprocessing and Statistical Analysis

To assess the impact of navigator‐based slice tracking on image quality and quantitative diffusion metrics, the coefficient of variation (CoV) was used as a metric of signal stability across the dynamic series. For each voxel, CoV was calculated as the ratio of the temporal standard deviation to the mean signal intensity across repetitions. Regions of interest (ROIs) were manually placed on the *b* = 50 image within the liver parenchyma by an experienced radiologist, avoiding large vessels and artifacts.

For each *b*‐value and each subject, the mean CoV across all voxels within the ROI was computed. Paired comparisons of average liver CoVs with and without NAV were performed using a two‐sided Student's *t*‐test. Statistical significance was defined as *p* < 0.05. Signal to noise ratio (SNR) in liver was calculated for ADC and trace images with *b* = 50 and 500. To measure the sharpness of the liver border full width at half maximum (FWHM) [22] was calculated for ADC and trace images with *b* = 50 and 500. Wilcoxon signed rank tests were performed to compare the SNR and FWHM of FB and NAV, respectively.

## 3 Results

**FIGURE 2:**
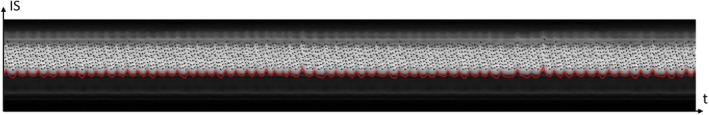
Respiration motion in a representative healthy subject. The navigator signal shows abdominal motion during respiration. The calculated motion information is overlaid as a red curve.

An example navigator signal acquired during free‐breathing is presented in Figure 2, clearly demonstrating periodic modulation corresponding to the subject's respiratory cycle. The projection‐based navigator provided a reliable surrogate for respiratory motion, with visible excursions aligning well with expected diaphragm displacement. Motion tracking information, derived from the navigator signal, was successfully overlaid on the signal trace, confirming the feasibility of using the navigator for real‐time slice tracking and motion correction.

**FIGURE 3:**
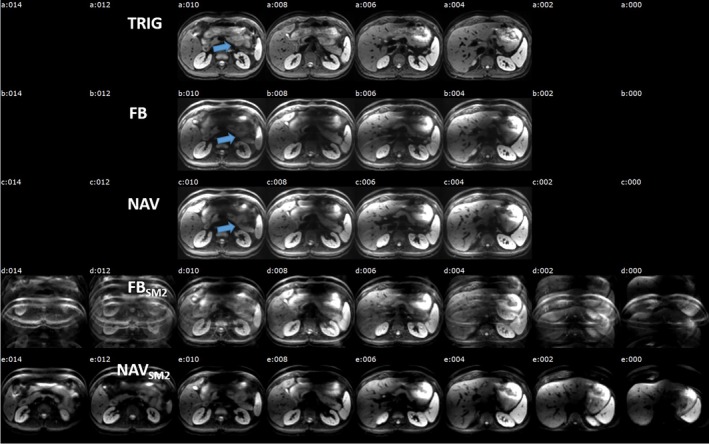
Representative trace images (*b* = 50 s/mm^2^) acquired with a vendor‐provided respiratory‐triggered (TRIG) sequence, navigator‐based slice tracking (NAV), and free‐breathing without motion correction (FB). Images were acquired using single‐band (8 slices) and multi‐band (16 slices) with multiband factor of 2 (SM2) excitations.

**FIGURE 4:**
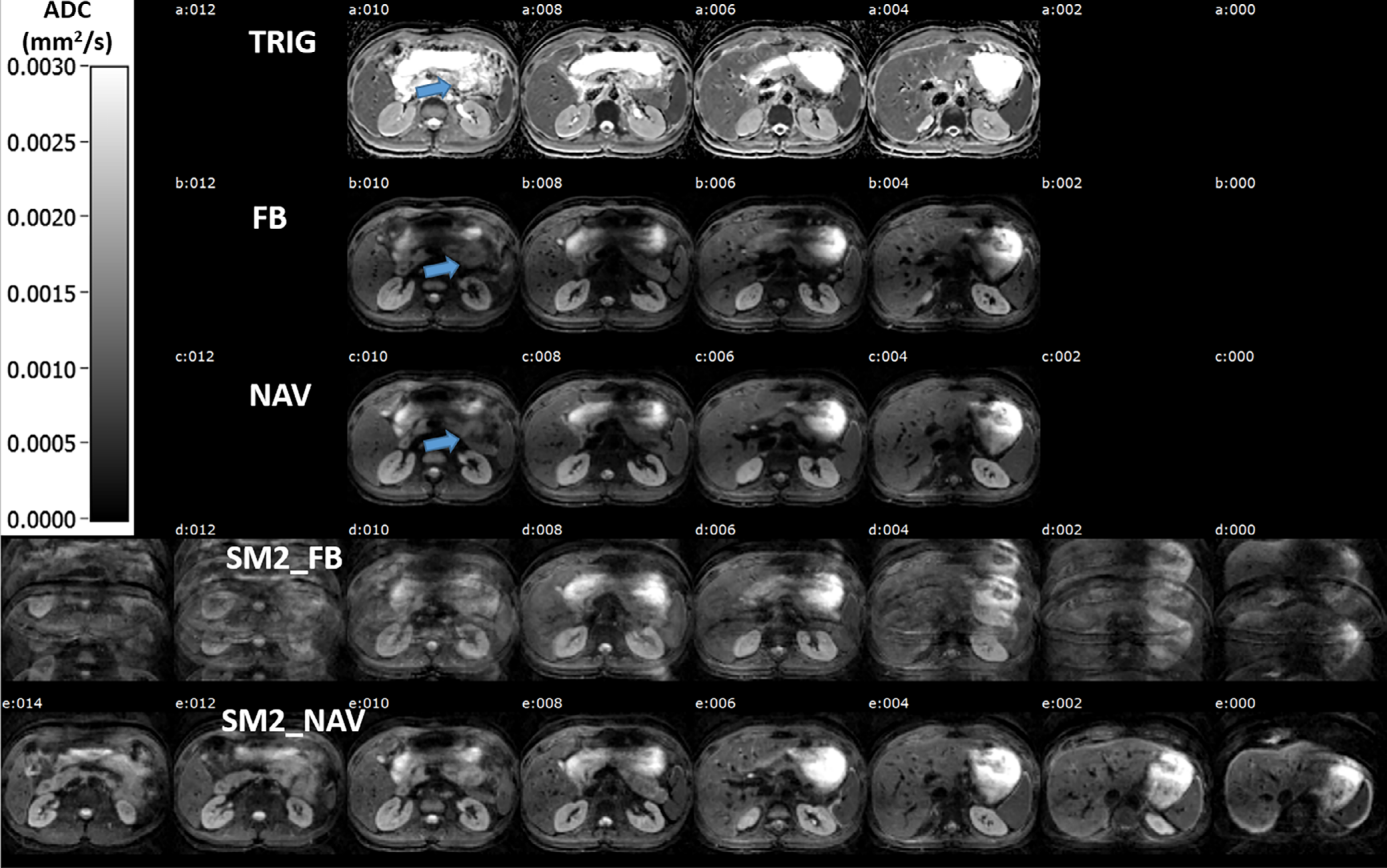
Representative ADC maps (mm^2^/s) acquired with a vendor‐provided respiratory‐triggered (TRIG) sequence, navigator‐based slice tracking (NAV), and free‐breathing without motion correction (FB). Images were acquired using single‐band (8 slices) and multi‐band (16 slices) with multiband factor of 2 (SM2) excitations.

**FIGURE 5:**
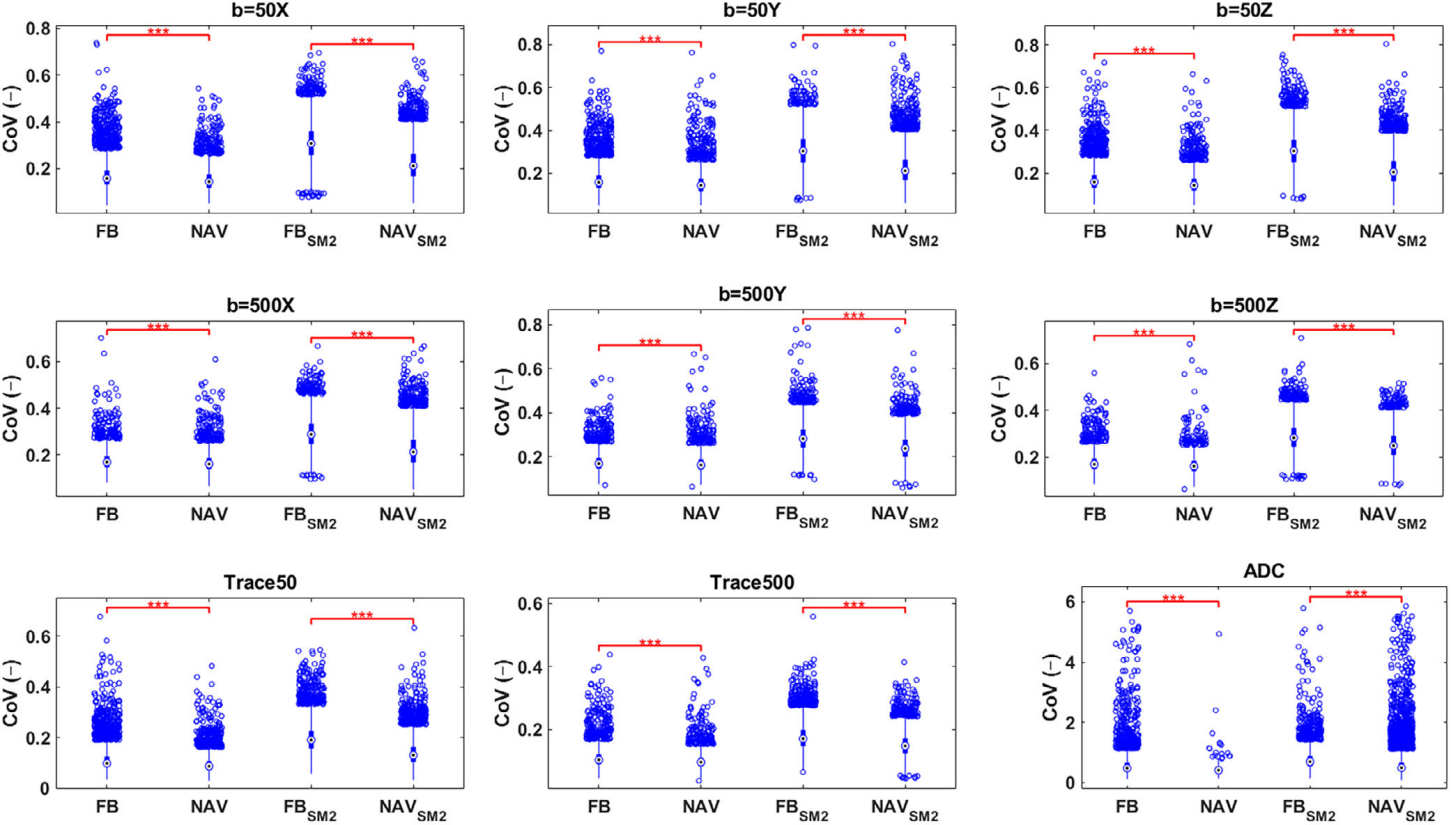
Corresponding coefficient of variation (CoV) in the liver from the same subject as in Figure 4. CoV values with NAV were significantly (*p* < 0.001) lower than without NAV (FB) in single‐band and SM2 cases.

Representative images are shown in Figure 3 (trace images, *b* = 50 s/mm^2^) and Figure 4 (ADC maps). Figure 5 presents the CoV for the trace images and ADC across repetitions for SB and MB acquisitions with (NAV) and without (FB) navigator correction. The vendor‐provided RT sequence does not provide dynamic trace images, and therefore CoV over time could not be calculated. ADC repeatability was assessed across 20 repeated acquisitions for SB and MB protocols with navigator‐based slice tracking. The CoV of ADC was significantly (*p* < 0.001) reduced with NAV compared to no‐NAV for both SB and MB acquisitions. While MB showed slightly higher variability than SB, the combined MB + NAV protocol demonstrated net improvement in ADC stability relative to MB without NAV (Figure 5). Nevertheless, visual inspection and quantitative ADC comparison indicate that the NAV‐based method achieves similar or improved image quality compared with the standard TRIG sequence, while maintaining predictable scan times.

**FIGURE 6:**
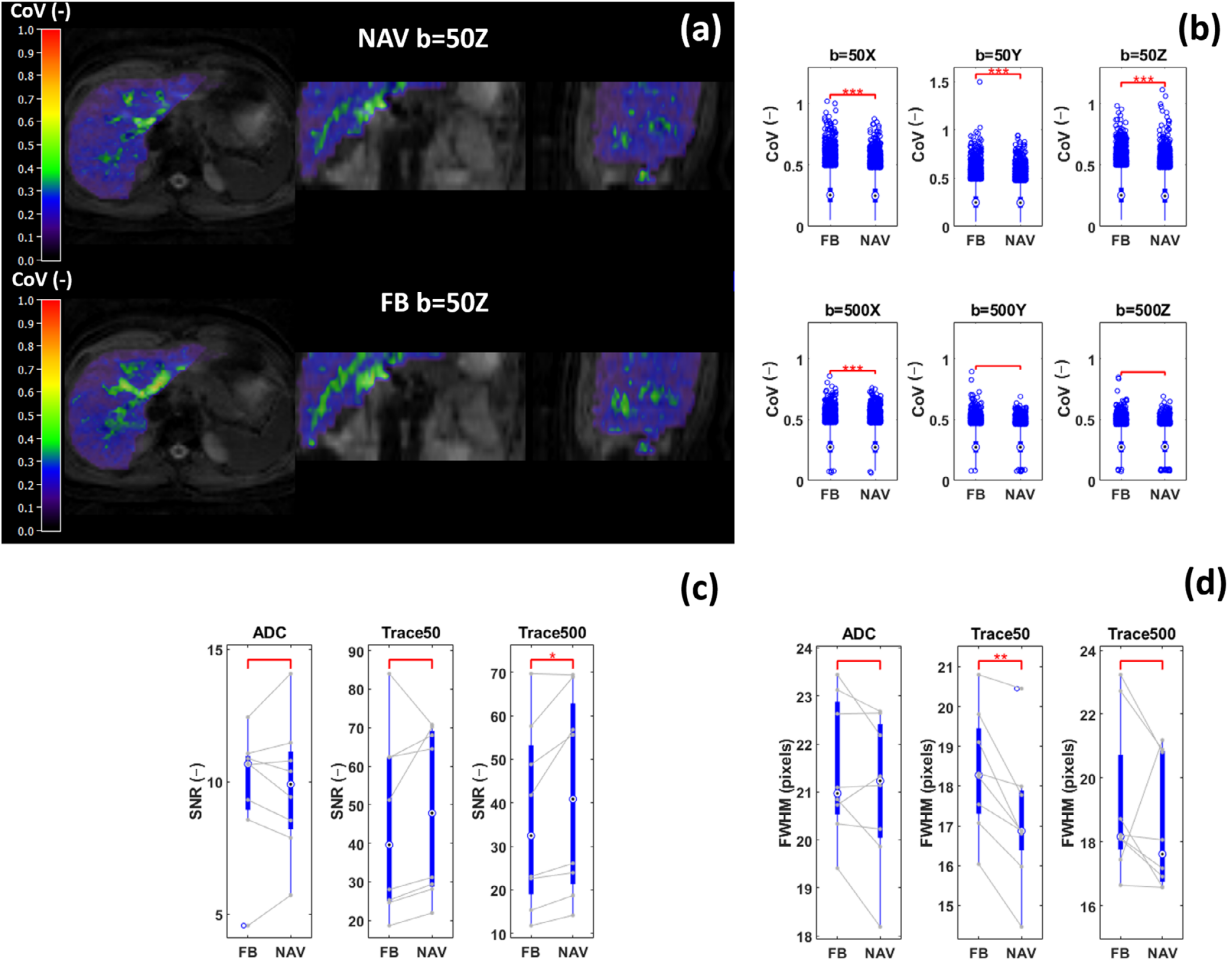
(a) Maps of the coefficient of variation (CoV) in the liver from a representative healthy volunteer. CoV values were extracted from the time series signal intensity variations in each liver voxel with (NAV) and without NAV (FB). One can see that NAV decreases CoV values. Differences in CoV for different *b*‐values of six subjects are shown in (b). The average CoV values with NAV were significantly (*p* < 0.001) lower than without NAV for *b* = 50*X*, 50*Y*, 50*Z*, and 500*X*. CoV were not significantly changed for *b* = 500*Y* and 500*Z* (*p* > 0.05). Differences in liver SNR values for ADC, trace image with *b* = 50 (Trace50) and *b* = 500 (Trace500) are shown in (c). The trends of SNR (gray lines) show increases when using NAV, especially significant in Trace500 (*p* < 0.05). Differences in liver sharpness (FWHM) values for ADC, and trace images with *b* = 50 (Trace50) and *b* = 500 (Trace500) are shown in (d). The trends of FWHM (gray lines) show decreasing FWHM (i.e., sharper images) when using NAV, especially significant in Trace50 (*p* < 0.01).

To quantitatively assess intrahepatic variability, CoV maps were generated for both acquisition schemes, as illustrated in Figure 6a. The NAV‐corrected images consistently showed lower CoV across the liver. A summary of the mean liver CoV values at each *b*‐value is provided in Figure 6b. On average, the liver CoV with *b* = 50 was significantly reduced when NAV was used (0.256 ± 0.086) compared to when it was not applied (0.262 ± 0.091), corresponding to a 2.3% reduction (*p* < 0.001). Although the absolute difference was modest, the statistical significance and consistent trend across subjects indicate improved measurement stability and reproducibility with NAV. The trend of the SNR showed an increase and FWHM showed a decrease (i.e., sharper images) using NAV. Specifically, the SNR of the trace image with *b* = 500 (*p* < 0.05) and FWHM of the trace image with *b* = 50 (*p* < 0.01) were significantly different.

**FIGURE 7:**
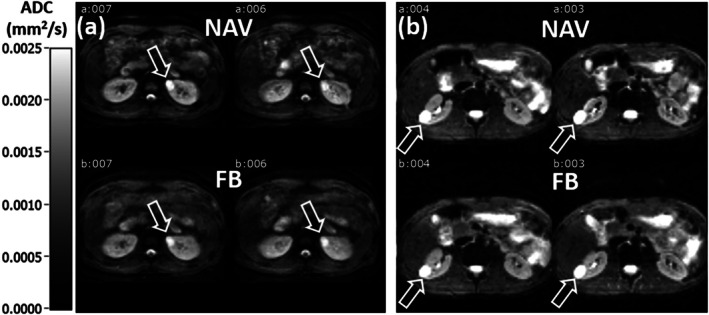
Impact of motion correction on liver ADC calculation in two subjects with abdominal cysts. When using NAV, cysts were clearly delineated against the surrounding liver parenchyma, enabling easier detection and more accurate lesion boundary definition. Without NAV during FB smearing and blurring such motion‐related artifacts were visible in ADC maps.

Figure 7 illustrates the differences in ADC maps between NAV and non‐NAV acquisitions in patients with abdominal cysts. With the use of NAV, cysts were clearly delineated against the surrounding liver parenchyma, enabling easier detection and more accurate lesion boundary definition. In contrast, non‐NAV acquisitions exhibited motion‐related artifacts, including smearing and blurring, which degraded organ borders and reduced confidence in anatomical interpretation. Images acquired with NAV showed markedly sharper organ contours, improved structural coherence, and fewer motion‐induced inconsistencies. As a result, more detailed and well‐defined anatomical structures were visible across multiple abdominal organs—including the liver, kidneys, and spleen.

**FIGURE 8:**
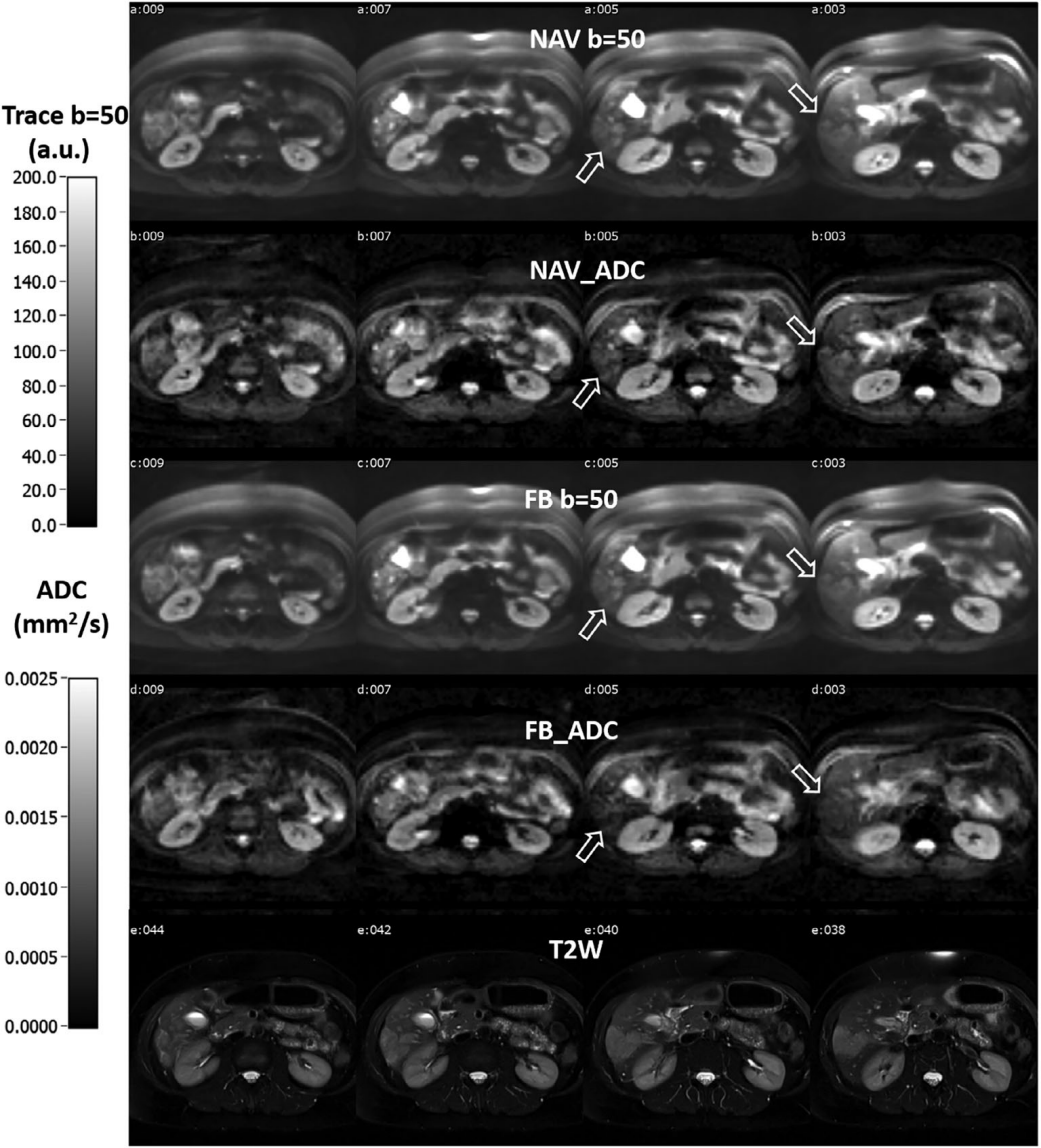
Impact of motion correction on trace images (*b* = 50 s/mm^2^) and liver ADC calculation in one subject with a hepatic tumor. The lesion margin was less blurred, and its shape and extent were better defined when using NAV.

The tumor patient also benefited from NAV‐enabled imaging (Figure 8). The liver mass was more conspicuous in both DWI and ADC maps when NAV was applied, showing a more accurate anatomical relationship with adjacent liver segments. In particular, the lesion margin was less blurred, and its shape and extent were better defined, which could aid in lesion characterization and volumetric assessment.

## 4 Discussion

In this study, we demonstrated the feasibility and efficacy of a navigator‐based slice tracking technique integrated with MB‐accelerated DWI using a single‐shot EPI readout. This approach enables real‐time compensation for respiratory motion, allowing robust liver DWI to be performed under free‐breathing conditions. Compared with conventional respiratory gating, the proposed method provides more efficient acquisition and improved motion robustness, representing an important step toward streamlined and reliable quantitative abdominal imaging.

Respiratory motion poses a major challenge in liver DWI due to the relatively long acquisition times. Traditional strategies to mitigate motion include respiratory gating and triggering, where image acquisition is restricted to a specific phase of the breathing cycle [23]. Although these techniques can improve image quality, they lead to longer and less predictable scan durations since a substantial portion of the respiratory cycle is discarded [24]. For example, the vendor‐supplied respiratory‐triggered sequence required approximately 6 min for the same imaging coverage, roughly double the scan time of our MB + NAV acquisition. In contrast, our navigator‐based approach integrates a short training period (32 navigator echoes) at the beginning of the scan, which adds only a few seconds of overhead. Slice tracking is then performed in real time throughout the acquisition, eliminating dead time and ensuring a predictable, shorter scan duration. This demonstrates that the MB + NAV approach can reduce scan time while maintaining image quality, compared with conventional respiratory‐triggered acquisitions.

To overcome this limitation, real‐time motion compensation methods have been explored. Tracking Only Navigator Echo (TRON), for example, employs a navigator echo to dynamically track diaphragm position and adjust slice location without relying on gating windows [25, 26]. In TRON, a 100‐mm long pencil‐beam excitation prepulse was placed at the interface between liver and lung. To mitigate signal loss on the navigator due to possible T_1_ saturation effects, anomalous dark bands from previous slice excitations are detected in the navigator and corrected by linear interpolation prior to calculation of positional change [24]. Comparing to TRON, we developed a simpler implementation using a single gradient‐echo projection readout as a navigator. Instead of interpolation, motion information was calculated after exclusion of the signal saturation in the navigator signal caused by EPI excitations [16]. This approach captures respiratory‐induced motion along the inferior–superior axis and eliminates the need for pencil‐beam designs. The projection navigator is easier to implement on standard clinical systems and integrates seamlessly into the DWI sequence without significantly increasing scan time. An additional practical advantage of the proposed NAV + MB method over TRON is its relative simplicity in clinical workflow. Unlike TRON, which requires dedicated sequence modifications and precise pencil‐beam placement, our method relies on standard navigator integration and automatically identifies the diaphragm position. This reduces the need for specialized programming or extensive technologist training, minimizes setup time, and lowers the risk of user error. Consequently, the method is more readily implemented in routine clinical practice while retaining the benefits of prospective motion correction.

Our results demonstrate the effectiveness of this method in reducing motion artifacts and improving image consistency. As shown in Figures 3, 4, 5, the inclusion of navigator‐based slice tracking resulted in more anatomically consistent images across slices (arrows). In addition, the corresponding ADC maps exhibited improved homogeneity in liver parenchyma, suggesting that the technique also enhances the stability of quantitative diffusion measurements. Although multiband acceleration improves temporal efficiency, it may increase sensitivity to motion and slice‐leakage artifacts. Our repeatability analysis demonstrates that navigator‐based slice tracking improves ADC stability for both SB and MB acquisitions; however, MB remains modestly more variable than SB (Figure 5). Therefore, the primary benefit of the proposed framework is improved robustness of accelerated DWI rather than a guarantee of superior quantitative precision compared to SB acquisitions.

A key quantitative finding was the significant reduction in the CoV of liver signal intensity across time points when NAV was used. This reduction, though modest (2.3%), was consistent and statistically significant across subjects (Figure 6b), underscoring the improved temporal stability and reduced motion sensitivity of the NAV‐enabled acquisition. These results are in line with previous studies employing real‐time slice tracking [16, 18, 19, 20], further validating the utility of this approach. Additionally, NAV correction was associated with improved signal‐to‐noise ratio (SNR) and image sharpness. The observed increase in SNR (Figure 6c) and decrease in FWHM (indicating sharper edges) (Figure 6d) with NAV suggests that motion correction effectively reduces blurring and noise amplification introduced by motion artifacts. Notably, the SNR improvement was statistically significant for the trace image at *b* = 500 (*p* < 0.05), while the FWHM reduction was significant for the trace image at *b* = 50 (*p* < 0.01). These findings demonstrate that NAV can preserve spatial resolution and improve image quality by minimizing motion‐induced degradation.

Although the proposed method visibly reduced motion artifacts and improved image sharpness, the quantitative improvement in CoV was smaller than that previously reported for kidney ASL (20%–25%) [16]. Several factors likely contribute to this difference. First, liver motion during respiration is more complex than kidney motion, involving not only superior–inferior translation but also substantial deformable motion, particularly in the anterior–posterior direction. Since the current navigator‐based approach primarily corrects rigid‐body translation, residual deformation‐related artifacts may persist, limiting the achievable reduction in CoV. Second, baseline variability in liver DWI was relatively low, especially for diffusion directions less sensitive to superior–inferior motion, reducing the potential for large relative improvements. Third, MB excitation increases temporal efficiency but can also increase sensitivity to motion and slice‐leakage artifacts. In the presence of deformable motion, these effects may partially counteract the benefits of slice tracking, particularly for higher MB factors. Despite the modest CoV reduction, the proposed method consistently improved visual image quality and sharpness, which are clinically relevant for lesion detection and anatomical delineation.

In the clinical cases involving two patients with abdominal cysts (Figure 7) and one with a known liver tumor (Figure 8), the application of NAV significantly enhanced both anatomical clarity and diagnostic quality in the diffusion‐weighted images. Compared to acquisitions without NAV, images acquired with NAV revealed more detailed and well‐defined structures across multiple abdominal organs—including the liver, kidneys, and spleen—despite being obtained under free‐breathing conditions.

The CoV reduction was significant for *b* = 500 in *X* direction, but not for the *Y* and *Z* directions. This does not imply a directional dependence of the proposed method itself, but rather reflects the interaction between respiratory motion and diffusion encoding: Respiratory motion in the abdomen is dominated by superior–inferior displacement, which primarily affects diffusion measurements acquired with gradients aligned along this direction. Consequently, motion‐induced variability is larger for the *X*‐direction encoding, allowing the benefits of slice tracking to be more clearly observed. In contrast, diffusion gradients applied in the *Y* and *Z* directions are less sensitive to respiratory motion, resulting in lower baseline variability and reduced statistical power to detect further CoV improvements. These findings indicate that the effectiveness of the proposed method is most apparent when motion sensitivity is high, while still providing consistent anatomical positioning across all diffusion directions.

Another limitation of the proposed navigator‐based slice tracking method is that it primarily compensates for rigid‐body translation, which dominates liver motion along the SI direction. Deformable motion, for example bending or compression of the liver along the anterior–posterior axis, is not directly corrected by slice tracking. Consequently, residual motion artifacts may persist in regions affected by organ deformation [27, 28]. In cases where deformable motion is significant, respiratory‐triggered acquisitions or phase‐selective techniques may provide complementary benefits, as they inherently acquire data at a specific respiratory phase, reducing artifacts due to shape changes. Future work could combine navigator‐based rigid motion tracking with deformable motion correction methods [29] to further improve image quality, particularly in patients with large or irregular liver motion patterns.

Although this study was conducted primarily in healthy volunteers, preliminary measurements in two patients indicated that navigator echo quality remained sufficient for reliable motion tracking, and no significant artifacts from susceptibility effects (e.g., bowel gas or tissue interfaces) were observed. Nevertheless, in larger patient populations or in regions with stronger susceptibility variations, navigator signal degradation could occur, potentially affecting motion estimation. Future studies should further assess the robustness of the NAV + MB method across diverse patients and consider strategies such as coil selection or signal averaging if local field inhomogeneities are present. Despite this theoretical limitation, our results suggest that the method is robust for correcting superior–inferior rigid‐body motion in both volunteers and patients.

The use of *b* = 500 s/mm^2^ was chosen to improve SNR and reduce motion sensitivity in free‐breathing body DWI, since the focus of this work was to examine the viability and accuracy of the proposed motion correction method. Although *b* = 800 s/mm^2^ can enhance diffusion contrast, it is more susceptible to noise and motion, which may increase variability in quantitative measurements.

An important advantage of our method is that it does not compromise acquisition time or require patient compliance with breath‐holding. This makes it particularly suitable for patients with limited breath‐hold capacity, such as those with chronic liver disease or other comorbidities. Furthermore, the method is compatible with MB acceleration, enabling high‐throughput DWI with adequate spatial coverage and resolution within clinically acceptable scan times.

Looking ahead, this technique holds promise for clinical applications where precise and reliable liver diffusion measurements are essential. Since ADC is a theoretically quantitative parameter for predicting therapeutic response at very early time points, stability of the underlying data from which it is calculated is essential. As to the high contrast, the detection of small hepatic lesions, especially in patients undergoing surveillance, would benefit from motion‐compensated DWI with enhanced image quality and quantitative reproducibility. Future studies should include larger patient cohorts with focal liver lesions to evaluate diagnostic performance and lesion conspicuity.

## 5 Conclusion

In this work, we demonstrated the feasibility of integrating navigator‐based slice tracking with multiband‐accelerated free‐breathing liver DWI. The proposed framework extends prior navigator‐based slice tracking methods to abdominal diffusion imaging, addressing respiratory through‐plane motion and multiband slice coupling. These results support the clinical translation of navigator‐based motion correction strategies to abdominal DWI and highlight their compatibility with modern accelerated diffusion imaging protocols.

## Funding

The authors have nothing to report.

## Data Availability

The data that support the findings of this study are available from the corresponding author upon reasonable request.
